# Genome-Wide Search for Eliminylating Domains Reveals Novel Function for BLES03-Like Proteins

**DOI:** 10.1093/gbe/evu161

**Published:** 2014-07-24

**Authors:** Shradha Khater, Debasisa Mohanty

**Affiliations:** Bioinformatics Center, National Institute of Immunology, New Delhi, India

**Keywords:** eliminylation, phosphothreonine lyase, fold-based function annotation, divergent evolution, genome context analysis, lanthionine synthetase

## Abstract

Bacterial phosphothreonine lyases catalyze a novel posttranslational modification involving formation of dehydrobutyrine/dehyroalanine by β elimination of the phosphate group of phosphothreonine or phosphoserine residues in their substrate proteins. Though there is experimental evidence for presence of dehydro amino acids in human proteins, no eukaryotic homologs of these lyases have been identified as of today. A comprehensive genome-wide search for identifying phosphothreonine lyase homologs in eukaryotes was carried out. Our fold-based search revealed structural and catalytic site similarity between bacterial phosphothreonine lyases and BLES03 (basophilic leukemia-expressed protein 03), a human protein with unknown function. Ligand induced conformational changes similar to bacterial phosphothreonine lyases, and movement of crucial arginines in the loop region to the catalytic pocket upon binding of phosphothreonine-containing peptides was seen during docking and molecular dynamics studies. Genome-wide search for BLES03 homologs using sensitive profile-based methods revealed their presence not only in eukaryotic classes such as chordata and fungi but also in bacterial and archaebacterial classes. The synteny of these archaebacterial BLES03-like proteins was remarkably similar to that of type IV lantibiotic synthetases which harbor LanL-like phosphothreonine lyase domains. Hence, context-based analysis reinforced our earlier sequence/structure-based prediction of phosphothreonine lyase catalytic function for BLES03. Our in silico analysis has revealed that BLES03-like proteins with previously unknown function are novel eukaryotic phosphothreonine lyases involved in biosynthesis of dehydro amino acids, whereas their bacterial and archaebacterial counterparts might be involved in biosynthesis of natural products similar to lantibiotics.

## Introduction

**Fig. 1.—:**
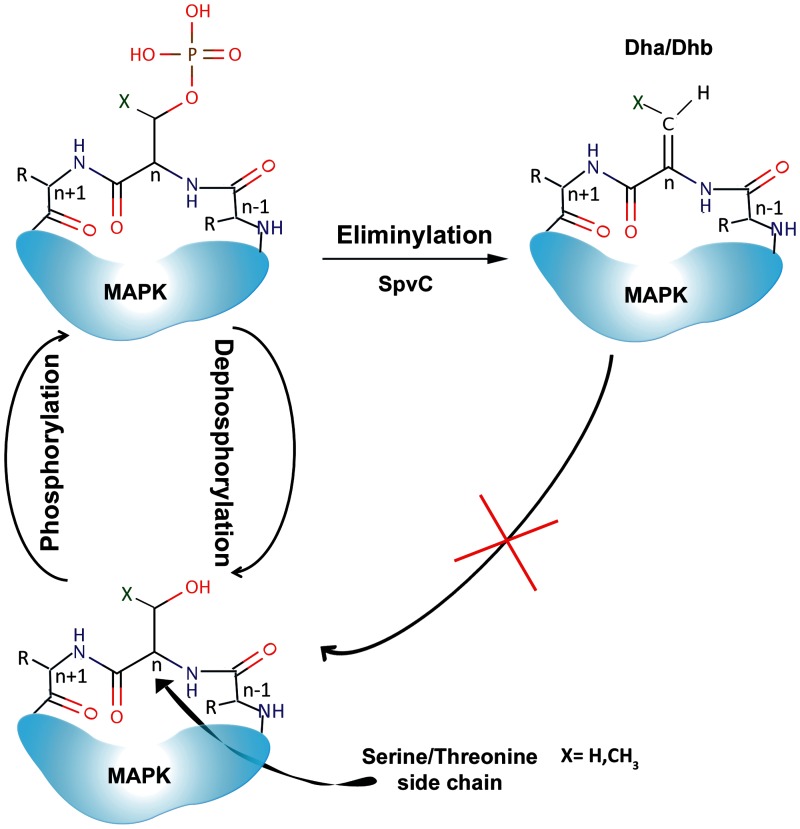
Schematic representation of eliminylation reaction.

Posttranslational modification (PTM) of proteins typically involves covalent attachment of a chemical moiety to side chains of amino acids in proteins. Eliminylation is a recently characterized PTM which irreversibly removes the phosphate group of phosphothreonine (pT) or phophoserine (pS) residues in phosphorylated proteins, thereby converting them to dehydrobutyrine (Dhb) or dehydroalanine (Dha), respectively (fig. 1). Eliminylation reaction is catalyzed by a newly discovered family of enzymes, namely Phosphothreonine lyases (PTLs). Unlike phosphatases which restore the hydroxyl moieties of Thr/Ser by removing the phosphate group, PTLs remove the phosphate group using β-elimination reaction involving Cβ–OP bond. Removal of phosphate group by β-elimination results in the formation of Cα = Cβ double bond (Li et al. 2007) and the hydroxyl group of Thr/Ser is not restored. In absence of the hydroxyl group eliminylated Thr/Ser cannot be phosphorylated again, thus the modification by eliminylation is irreversible.

Eliminylating enzymes were recently discovered in three different pathogenic bacteria. It was shown that *Shigella* OspF (Li et al. 2007), *Pseudomonas* HopAI1 (Zhang et al. 2007) and *Salmonella* SpvC (Mazurkiewicz et al. 2008) dephosphorylate pT of pT-X-pY motif in dual phosphorylated, fully active host MAPKs (mitogen-activated protein kinases). This inactivates MAPKs in the host, hindering the initiation of innate immune response and thus increasing the chances of survival of these pathogens. Structural and biochemical analyses have provided novel insight into the catalytic mechanisms of PTLs (Zhu et al. 2007; Chen et al. 2008). Crystal structures of SpvC-like PTL from different organisms, such as *Salmonella typhimurium* [Protein Data Bank (PDB): 2Z8N, 2Z8M, 2Z8O, 2Z8P] (Chen et al. 2008), *Chromobacterium violaceum* [PDB: 3BO6], *Sa. enterica* [PDB: 2P1W, 2Q8Y] (Zhu et al. 2007), and *Shigella flexneri* [PDB: 3I0U] have shown that it has a novel α/β-fold. The binding mode of SpvC-like PTL to its substrate was revealed by crystal structures [PDB: 2Q8Y and 2Z8P], where substrate peptide was cocrystallized along with SpvC-like PTL (Chen et al. 2008). The substrate-bound structure showed that pY of pT-X-pY motif was essential for substrate recognition and the positively charged pocket that binds pY is distinct from the pocket that binds and eliminylates pT (Zhu et al. 2007; Chen et al. 2008).

More recently, PTL catalytic activity has also been discovered in type IV lantibiotic synthetases involved in biosynthesis of lantibiotics in bacteria (Goto et al. 2010, 2011). Lantibiotics are posttranslationally modified bioactive peptides containing characteristic lanthionine (Lan) or methyllanthionine (MeLan) groups. Lantibiotics are synthesized by four classes of lantibiotic synthetases (Goto et al. 2010). VenL-like multifunctional enzymes, belonging to type IV lantibiotic synthetases (LanL), harbor three distinct functional domains and catalyze biosynthesis of lanthipeptides using Ser/Thr- and Cys-rich ribosomal polypeptides as substrates. Interestingly, bioinformatics analysis of LanL-like multidomain enzymes suggested the presence of Ser/Thr kinase-like domain, C-terminal cyclase domain, and a novel N-terminal domain that shared weak homology with SpvC-like PTLs. The catalytic residues of PTL (Goto et al. 2010, 2011) were conserved in this N-terminal domain, thus suggesting putative lyase activity. Together these results suggested the possible role of LanL in biosynthesis of lanthipeptides using a new mechanism. Subsequent experimental studies confirmed PTL activity in the N-terminal domain of VenL and led to the discovery of novel type IV biosynthetic pathway for lanthipeptides. The kinase domain of LanL-like proteins first phosphorylates a Ser/Thr residue on Ser-, Thr-, and Cys-rich precursor peptide and this is followed by eliminylation of phosphate group by lyase domain converting pT or pS to Dhb or Dha, respectively. C-terminal cyclization domain subsequently catalyzes formation of Lan or MeLan moieties by covalent attachment of Dha or Dhb residues to cystines.

Discovery of type IV lantibiotic synthetases has established that PTL domains are not only present in bacterial virulence factors but also in bacterial biosynthetic pathways. However, straightforward sequence similarity searches indicate that both these families of lyases are restricted to bacteria only. Brennan et al. have hypothesized that eliminylating enzymes might be involved in eukaryotic signaling (Brennan and Barford 2009). Even though until now there are no reports of PTL homologs in eukaryotes, crystallin in aging human lenses was found to contain Dha (Srivastava et al. 2004) and the crosslink formed by Dha and lysine/cysteine is also present in human lenses (Linetsky et al. 2004). However, enzymes which catalyze formation of these dehydro amino acids in crystallin are not known. In view of the presence of large number of protein kinases in human genome, it is tempting to speculate that formation of these dehydro amino acids might be catalyzed by type IV lantibiotic-like pathways involving kinase and phosphothreoine lyase activity. Interestingly, LanC-like proteins are also present in humans and certain other eukaryotes, though their function is yet unknown (Brennan and Barford 2009). It is possible that LanC-like enzymatic domains act on Dha/Dhb and crosslink them to cysteine or lysine residues. These evidences encouraged us to systematically search for putative PTL-like enzymes in humans and other eukaryotes.

In this work, we have carried out a systematic genome-wide search for PTL enzymes in humans and other eukaryotes. Apart from sensitive profile-based searches, using the crystal structures of bacterial pathogenic PTLs, we have also carried out search for proteins which can potentially adopt PTL fold. Structure-based analysis revealed that BLES03 (basophilic leukemia-expressed protein 03), a human protein with unknown function, has PTL fold and it also shares catalytic residues of known PTLs. Our analysis also revealed presence of BLES03-like proteins in archaea. The resemblance of the genomic neighborhoods of archaeal BLES03-like enzymes to other catalytic components of LanL pathway also suggests PTL-like function for BLES03 homologs in archaea. Hence, a completely independent line of evidence based on synteny also suggests PTL activity for BLES03. Interestingly, our genome-wide search has led to the discovery of putative PTL-like proteins in all three branches of life, that is, bacteria, archaea, and eukaryotes. We also consider the evolutionary relationship between these families, whose sequences are so diverged that even most sensitive sequence-based method did not find any relation, yet their overall three-dimensional fold and active site pocket have been conserved to perform same function. Association of BLES03 with many different cancer types and known role of bacterial PTLs in disruption of MAPK signaling also suggest that our in silico prediction of PTL activity for BLES03 might provide clues for deciphering molecular basis of the disease association of BLES03.

## Materials and Methods

### Sequence- and Structure-Based Search for New Eliminylating Domains

In order to identify novel eliminylating domains in various genomes, the experimentally characterized eliminylating domains [Swiss-Prot: P0A2M9, F2R8I9 and Q9H3H3] were used as query and sequence similarity searches were carried out in nonredundant (nr) database (released in September 2012) of National Center for Biotechnology Information (NCBI). Same searches were also carried out on current version of nr database using the web version of BLAST tool. Though the number of hits has increased, overall taxonomical distribution of the families remained same. For VenL [Swiss-Prot: F2R8I9], sequence of the fragment containing only the PTL domain (amino acid range 1–170) was used as query. Pairwise sequence similarity searches were carried out using a local version of NCBI BLASTp (Altschul et al. 1997; Johnson et al. 2008) and PSI-BLAST program was used for iterative profile-based searches. The *E* value cutoff of 10^−^^3^ was used for both BLASTp and PSI-BLAST. First round of PSI-BLAST uses BLAST to search for proteins similar to query in a given database. For subsequent rounds, it builds a profile from the hits obtained in previous round and queries the target using this profile. The sequence of events was repeated till no new sequences could be added to the profile, that is, till convergence. Profile–profile comparisons were done using HHPred server (Soding et al. 2005). In addition to comparison of profiles built using two sequences, it also compares their secondary structures. To assess the significance of results, HHPred reports *E* value and probability. *E* value does not incorporate secondary structure score, whereas probability contains contribution from secondary structure score. PDB70 and PfamA databases of HHPred were used as target for remote homology detection. PDB70 database contains profiles built using representative sequences present in PDB database. The sequences are chosen such that no two sequences have identity above 70%. PfamA database is derived using multiple sequence alignment of all PfamA families from Pfam database. Multiple sequence alignment (MSA) for each family was built using ClustalW. For phylogenetic analysis, bootstrapped trees were built and visualized using QuickTree (Howe et al. 2002) tool of PHYLIP package and iTOL, respectively. The phylogenetic trees can be accessed using the URL http://itol.embl.de/shared/shradha (last accessed July 30, 2014). Also the Newick files of all three trees have been included in supplementary files S3–S5, Supplementary Material online, for detailed analysis. The leaves of phylogenetic trees are colored based on the taxonomical classes and the text on branches indicates bootstrap values using 1,000 replicates. Aligning sequences from the distantly related four families was complicated. In order to increase the quality of alignment for such highly divergent proteins, structural information has been used by PROMALS-3D software to guide the MSA (Pei et al. 2008). As no crystal structures were available for LanL-like proteins, representative sequences of LanL-like proteins VenL and RamC were given as input along with crystal structures of BLES03 and SpvC-like PTL to obtain the MSA (fig. 5).

DaliLite program from Dali server (Holm and Rosenstrom 2010) was used to search for the structural neighbors of the eliminylating domains in PDB by using the structure 2Q8Y of known eliminylating domains as query. DALI Z score above 2 indicates statistically significant structural similarity. Superimposition, analysis, and visualization of the structures were carried out using academic version of PyMOL software The PyMOL Molecular Graphics System, Version 0.99 Schrödinger, LLC. PROMALS3D (Pei et al. 2008) software was used for structure-based MSA of representative sequences and structures of known and predicted eliminylating domains. PROMALS3D has advantage over conventional MSA software because it uses structural information and predicted secondary structure to guide the MSA of distantly related homolog. PROMALS3D employs PSIPRED (McGuffin et al. 2000) algorithm to predict secondary structure for those proteins for which three-dimensional structures are not available. In addition to PROMALS3D, secondary structure predictions were also carried out for VenL-like proteins using JPRED server (Cole et al. 2008). The results from JPRED were compared with consensus secondary structure predicted by PROMALS3D and available crystal structures of SpvC-like PTL, BLES03, and eIF4E. Schematic diagrams of pT- and phosphotyrosine-binding pocket interactions were generated using LigPlot+ (Laskowski and Swindells 2011).

### Modeling of BLES03-Peptide Complex

In order to model the substrate peptide in complex with BLES03, flexible docking approach was used and the peptide fragment QYFMpTE from ERK (extracellular-signal regulated kinases) was docked in the catalytic site of BLES03 using HADDOCK server (de Vries et al. 2010). During the docking process, apart from the substrate peptide ligand, the loop region on the BLES03 receptor which contained R215 and R169 was also made flexible. HADDOCK outputs top ten clusters ranked by HADDOCK score, which includes various energy terms. As all the top ten complexes had ERK peptide docked in the active site pocket predicted by us, the cluster where pT was at a distance of 6 Å or less from K128, K204, K159, and Y175 was chosen. The conformation with minimum energy in the chosen cluster was considered for further analysis.

### Molecular Dynamics Simulations on BLES03-Peptide Complex

It may be noted that HADDOCK permits only a limited number of residues to remain flexible during docking. To incorporate complete flexibilities in both ligand and the receptor, explicit solvent molecular dynamics (MD) simulations were carried out on the complex obtained from HADDOCK. The peptide bound BLES03 was solvated in a rectangular box of TIP3 water molecules (Jorgensen et al. 1983) such that box boundaries were at a distance of 10 Å from the outermost atoms of the BLES03-peptide complex along *x*, *y*, and *z* directions. MD simulations were carried out using AMBER11 package (Case et al. 2010) and ff03 force field (Duan et al. 2003). Force field parameter assigned for the pT residue in the substrate peptide was based on earlier work by Homeyer et al. (2006). The initial structure of the solvated BLES03-peptide complex minimized using steepest descent approach to a RMS gradient of 0.001 kcal/mol/Å using SANDER module. After minimization, MD runs were carried out by SANDER module using a time step of 1 fs and bonds involving hydrogen atoms were constrained using SHAKE (Ryckaert et al. 1977). Long-range electrostatic interactions were computed using Partile Mesh Ewald (Darden et al. 1993) approach and a cutoff of 10 Å was used in the direct space for nonbonded interactions. Before starting the production dynamics, a two-stage equilibration process was carried out. In the first stage, simulations were carried out in NVT ensemble and the temperature of the system was gradually raised from 100 to 300 K over 5 ps of simulation time. The temperature of the system was constrained to the specified value by using Langevin dynamics temperature coupling scheme and collision frequency of 5 ps^−^^1^. After the temperature of the system reached 300 K, the second stage of equilibration was carried for 15 ps using NPT ensemble to adjust the density of water to 1 g/cc. After the system had attained a temperature of 300 K and density of 1 g/cc, production run was carried out for 30 ns in NPT ensemble using PMEMD (Partile Mesh Ewald Molecular Dynamics) module of AMBER.

As the distance between R169 and pT219 was approximately 14 Å, the cutoff was increased to 16 Å for a short period between 17 and 21 ns of simulation time. Indeed, this was the period in which R169 came closest to pT219. The distance between R169 and pT219 in various conformations sampled during the MD simulations was calculated using ptraj module of AMBER and other in-house PERL scripts. In order to analyze the solvent accessible surface area of pT, coordinates were extracted from the trajectory at an interval of 100 ps in PDB format using ptraj module and provided as input to the NACCESS program (Hubbard and Thornton 1993).

### Synteny of BLES03 and LanL-Like Proteins

Contextual information was extracted from fully sequenced prokaryotic genomes using in-house Perl scripts. Five genes from upstream and five from downstream were picked. When completely sequenced genomes were not available Gene database from NCBI was queried. In addition to records from fully sequenced genomes, intermediate genomic records from unfinished genomic data are also included in Gene database. Functional information about neighboring genes was extracted using Pfam database (Finn et al. 2014).

## Results

### Sequence-Based Search for PTLs

**Fig. 2.—:**
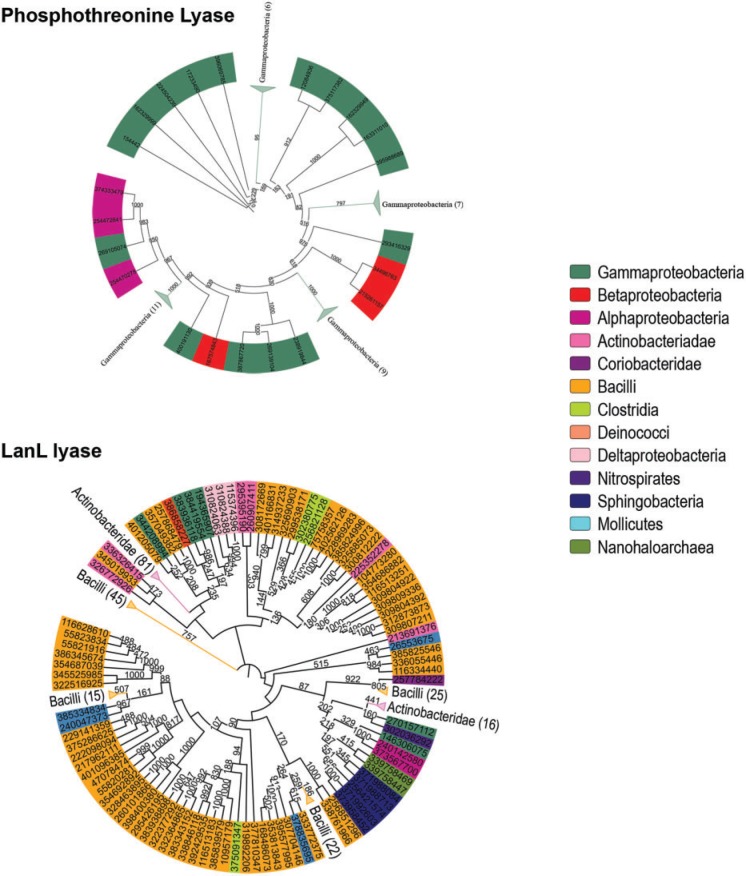
Phylogenetic tree of SpvC-like PTL and VenL-like lyase. Phylogenetic trees built using SpvC-like PTL (top) and VenL-like lyase (bottom). Color on outer ring indicates taxonomic classes of the organism to which the respective protein belongs. To improve clarity, clades containing proteins from same taxonomical classes were collapsed and labeled. The numbers in bracket indicate number of sequences contained in that clade. The numbers next to branches indicate number of time-associated branches clustered together in a bootstrap test with 1,000 replicates.

Pairwise BLASTp (Johnson et al. 2008) and profile-based PSI-BLAST (Altschul et al. 1997) searches using SpvC sequence as query yielded a total of 53 and 55 proteins, respectively (supplementary file S1, Supplementary Material online). All these proteins showed a sequence similarity in the range of 41–100% to SpvC with query coverage above 34%. Phylogenetic distribution of PTL sequences obtained by BLAST and PSI-BLAST searches was restricted to pathogenic bacterial species (supplementary table S1 in supplementary file S2, Supplementary Material online) (fig. 2*A*). In contrast to SpvC-like PTLs, PSI-BLAST search for sequences similar to lyase domains of VenL-like proteins resulted in 301 hits (supplementary table S1 in supplementary file S2 and supplementary file S1, Supplementary Material online). These 301 proteins showed a sequence similarity in the range of 41–100% to PTL domain of VenL with query coverage above 24%. The distribution of these VenL-like PTL domains was also restricted to bacterial families (fig. 2*B*). Although VenL-like proteins are present in diverse bacterial classes such as actinobacteria, proteobacteria, bacilli, sphingobacteria, and mollicutes, the distribution of SpvC-like PTLs is restricted to alpha, beta and gamma classes of proteobacteria. There was no overlap between sequence neighbors of SpvC- and VenL-like PTL domains, indicating high sequence divergence between these families despite similarities in catalytic activity. Structure-based profile–profile comparison tool, HHPred (Soding et al. 2005), was used to search the Pfam database (Punta et al. 2012) for protein families that are similar to SpvC- and VenL-like PTLs. Interestingly with both SpvC and VenL as query, top hit was SpvC-like PTL family [Pfam ID: PF03536] (supplementary table S2*A* in supplementary file S2, Supplementary Material online). Similarly, when HHPred search was carried out in PDB70 database (Soding et al. 2005) using SpvC- and VenL-like PTL domains as query, the crystal structures of SpvC-like PTLs were obtained as top hits (supplementary table S2*B* in supplementary file S2, Supplementary Material online). Though an evolutionary link could be established between SpvC- and LanL-like PTLs, BLASTp, PSI-BLAST and HHPred could not detect any new families of PTL.

### Structure-Based Search for Remote Homologs Reveals PTLs Function for BLES03

**Fig. 3.—:**
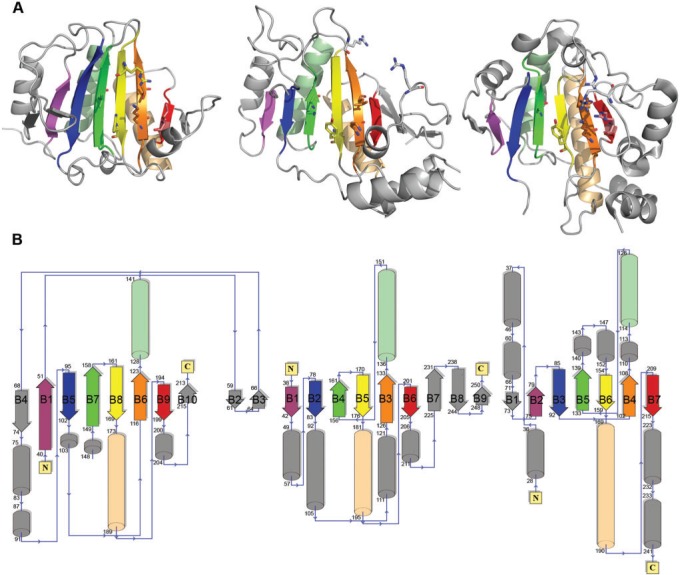
Structural similarity of eIF4-E, BLES03, and SpvC-like PTLs. (*A*) Cartoon representation of eIF4-E (left), BLES03 (center), and SpvC-like PTL (right). Catalytic residues in SpvC and the residues of eIF4-E and BLES03 which align with catalytic residues of SpvC are shown in stick representation. Conserved core is shown in rainbow colors. Corresponding core secondary structures in ribbon and topology diagrams (*B*) have same colors. (*B*) Topology diagrams generated using PDBSUM.

It is well known that there are several enzyme families, which show a conserved structural fold despite high degree of sequence divergence (Furnham et al. 2012). Hence, structural similarity searches were carried out in PDB using Dali Server (Holm and Rosenstrom 2010) to identify sequences that adopt unique α/β-fold of PTLs (Ke et al. 2011). Table 1 shows results of Dali search ranked by *Z* score when the PTL structure 2Q8Y was used as a query. Apart from other known PTL structures, eukaryotic translation initiation factor 4E (eIF4-E) [PDB: 2IDR] and human basophilic leukemia-expressed protein (BLES03) [PDB: 2Q4K] have RMSD values less then 4 Å and *Z* score above 5, indicating statistically significant structural similarity with PTL. Although structural similarity between BLES03 and eIF4-E has been reported by SCOP (Andreeva et al. 2008), their similarity to PTLs was not reported earlier. Figure 3 shows the structural fold and topology diagram for representative structures belonging to PTL, eIF4-E, and BLES03 families. As can be seen from figure 3*A*, the core region consisting of an array of seven beta strands and two helices is conserved in all three different structures in terms of their relative orientation and packing. However, in one-dimensional sequence the order in which these structurally equivalent segments are connected is different (fig. 3*B*). Hence, sequence/profile-based methods such as PSI-BLAST or Pfam failed to detect any similarity between these three protein families. However, structure-based search could identify similarity between SpvC-like PTL, eIF4-E, and BLES03. It is interesting to note that among these three protein families which adopt a common fold, functions are known for PTL and eIF4-E (Matsuo et al. 1997). The native function of eIF4-E is to bind m7G cap at the 5′-end of mRNA and thereby help in recruiting ribosomes to mRNA (Sonenberg 2008). However, BLES03 is essentially a protein with unknown function. Therefore, to explore whether BLES03 shares catalytic residues with PTL or eIF4-E we analyzed the putative active site pocket of BLES03.

**Table 1:** Results of Structural Similarity Search Using DALI

Query	Hits	DALI *Z* Score	RMSD	Nalign	% Sequence Similarity	Family
2Q8Y	2Z8P	39.1	0.4	213	100	PTL
	3BO6	35.8	1.1	213	80	PTL
	3I0U	30.7	1.1	191	93	PTL
	2IDR-A	5.8	3.6	114	11	eIF4E-1
	2Q4K-A	5.7	3.8	122	7	C11ORF68
	1ZTP-A	5.6	3.3	120	7	C11ORF68
	3NOG-B	4.7	4	107	6	Acriflavin resistance protein
	3DQ0-A	4.6	3.8	116	9	Cytokinin dehydrogenase

**Fig. 4.—:**
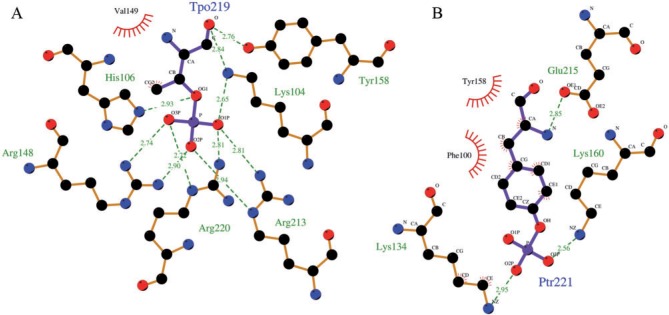
pT- and pY-binding pockets in SpvC. Schematic depiction of pT (*A*) and phosphotyrosine (*B*) binding pocket in SpvC crystal structure [PDB: 2Q8Y]. For clarity, only interactions with phosphorylated residues are shown. The plot was generated using LigPlot+ (Laskowski and Swindells 2011).

**Fig. 5.—:**
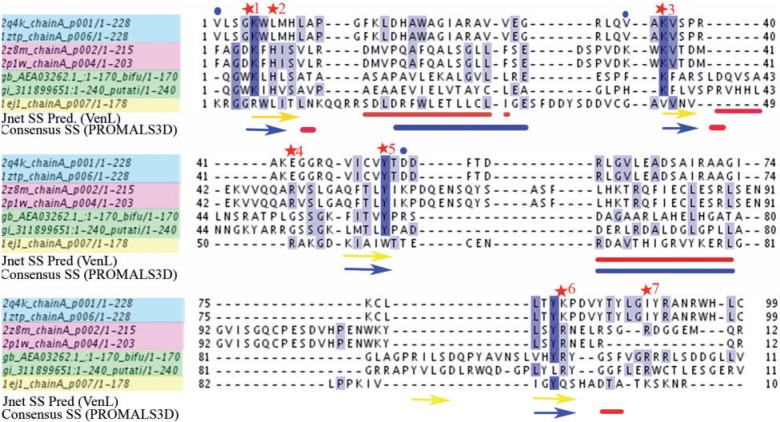
Structure-based sequence alignment provides a link between BLES03, SpvC, and LanL lyase. Structure-based sequence alignment of BLES03 structures (highlighted in blue), SpvC-like PTL structures (in pink), structure of eIF4-E (in yellow), and sequences of LanL-like proteins (in green). Red star: active site residues of SpvC-like PTL; blue circles: phosphotyrosine (pY)-binding residue. Consensus secondary structure and predicted secondary structure of VenL’s lyase domain are shown below the alignment. Numbered stars provide a link to same residues in figure 6*A*.

**Fig. 6.—:**
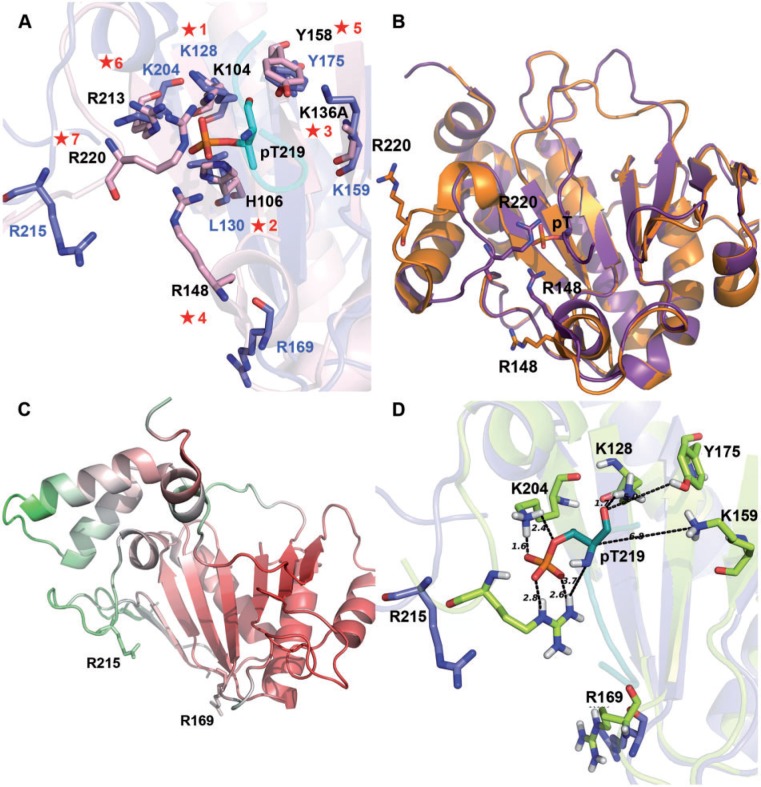
Comparison of the active site pockets of SpvC and BLES03. (*A*) Active site of SpvC-like PTL (pink) and putative active site of BLES03 (blue). Numbered stars provide a link to corresponding residues in the multiple sequence alignment shown in figure 5. pT from MAPK peptide is shown in green. (*B*) In SpvC, conformational changes bring R220 and R148 into the active site pocket on substrate binding. SpvC (orange), SpvC with substrate (purple). (*C*) 1ZTP (BLES03) colored according to its B-factor (red: low, white: medium, green: high B-factor). Loops containing R169 and R215 have medium and high B-factor, respectively, making it likely that they can move into the interior on substrate binding. (*D*) Truncated ERK peptide (QYFMpTE) was docked on to BLES03 structure 2Q4K. Blue: residues before docking, light green: residues after docking, dark green: docked pT. Dashed lines show distance between interacting atoms. Conformational changes similar to SpvC are seen in BLES03 on docking—loop containing R215 moved into the catalytic pocket.

Structural and biochemical analyses have elucidated catalytic mechanism of SpvC-like PTLs (Zhu et al. 2007; Chen et al. 2008; Ke et al. 2011). The crystal structure of PTL from *Sa. enterica* in complex with the substrate peptide (QYFMpTEpYVA) from human ERK revealed that active site of PTL consists of a phosphotyrosine-binding pocket in addition to the pT-binding pocket (fig. 4). The phosphate moiety of pY is located in a positively charged groove composed of K134, K160, and F100 (fig. 4*B*) (Zhu et al. 2007). The binding of this phosphate helps in orienting the pT moiety of the substrate into the catalytic pocket of the enzyme (Li et al. 2007). The phosphate group of pT makes strong ionic interactions with side chains of K104, R148, R213, and R220 (fig. 4*B*). These interactions lock pT in the catalytic pocket and also prevent its hydrolysis. K104, K136, H106, and Y158 are involved in catalysis. K136 abstracts an α-proton of pT creating a carbanion intermediate which is stabilized by K104 and Y158 (Zhu et al. 2007; Chen et al. 2008; Ke et al. 2011). H106 protonates oxygen atom of the leaving phosphate group and Y158 keeps K136 in a deprotonated state, which is essential for its role as a catalytic base (Zhu et al. 2007). Figure 5 shows the conservation profile of these catalytic and binding pocket residues in the structure-guided multiple sequence alignment (MSA) for the four families, that is, SpvC-like PTL, lyase domain of LanL-like proteins, BLES03, and eIF4-E. The comparison of secondary structure of the LanL-like proteins predicted by Jnet algorithm (Cole et al. 2008) and consensus secondary structure predicted by PROMALS3D revealed that they are highly likely to share the conserved structural fold of PTLs. As can be seen from figure 5, pY-binding residues were not conserved in LanL, BLES03, and eIF4-E. Earlier studies which used sequence-based alignments could not find residues corresponding to R213 and R220 in LanL (Goto et al. 2010, 2011). Structure-guided sequence alignment (Pei et al. 2008) revealed that all seven active site residues were conserved (fig. 5) in LanL, consistent with experimentally verified PTL activity of these enzymes in lanthipeptide biosynthetic pathways. eIF4-E showed conservation of only three of seven pT-binding residues. Putative catalytic residues corresponding to K136 and H106 were also not conserved. This explains lack of PTL activity of eIF4-E despite sharing a structural fold similar to PTLs. Interestingly, BLES03 had two of the three catalytic residues absolutely conserved. The residues corresponding to K136 and Y158 of PTL corresponded to K159 and Y175, respectively, in BLES03. A closer look at the superimposed structures of PTL and BLES03 revealed that amino group of K204 (BLES03) was closest to the leaving oxygen atom (fig. 6*A*) and K204 could play the role of H106 of PTL. Several earlier studies have demonstrated that Lys can indeed protonate leaving groups (Stein and Geiger 2002; Castro et al. 2009; Ganguly et al. 2009). It has also been shown that upon H106K mutation, PTL retains partial activity (5- to 7-fold decreased *K*_cat_ value) in transferring a proton to the leaving group (Zhu et al. 2007) and its *K*_m_ value is comparable to the wild-type enzyme. The residue corresponding to K204 in SpvC-like PTL is R213 which is a weaker proton donor compared with lysine. Out of the four amino acids K104, R148, R213 and R220 that comprise pT-binding residues in SpvC-like PTL, residues equivalent to K104 and R213 were also conserved in BLES03 (i.e., K128 and K204, respectively). As discussed later, structural counterparts of pT-binding residues, R148 and R220, were also found to be present in BLES03. Hence, structural fold-based comparison indentified novel PTL which even the most sensitive sequence-based method could not detect.

### MD Simulations Reveal Ligand-Induced Conformational Changes in BLES03 Similar to PTLs

The fold level structural comparisons and analysis of active site residues suggested that BLES03 has two out of the three catalytic residues and two out of the four pT-binding pocket residues conserved. Conformational changes upon binding of substrate have been observed in SpvC which brings R220 and R148 into the catalytic pocket (Chen et al. 2008) (fig. 6*B*). This prompted us to search for residues on loop regions of BLES03 that could be involved in recognition of pT and help in its PTL enzymatic activity. Based on structural superposition of BLES03 and PTL crystal structures, R215 and R169 were identified as probable residues in BLES03 that could flip into the catalytic pocket once substrate is bound. Interestingly, B-factor of loop containing R215 and R169 was higher than rest of the protein (fig. 6*C*). In order to test this hypothesis, the pT-containing peptide fragment QYFMpTE from ERK, native substrate of SpvC-like PTL, was docked onto BLES03 structure using HADDOCK web server (de Vries et al. 2010). Top ten high scoring clusters from HADDOCK web server showed that the ERK peptide always docked into the predicted catalytic pocket (supplementary fig. S1*B* in supplementary file S2, Supplementary Material online). In majority of these clusters, R215 also showed a movement toward the catalytic pocket (fig. 6*D* and supplementary fig. S1*A* in supplementary file S2, Supplementary Material online). However, R169 did not show any significant movement.

**Fig. 7.—:**
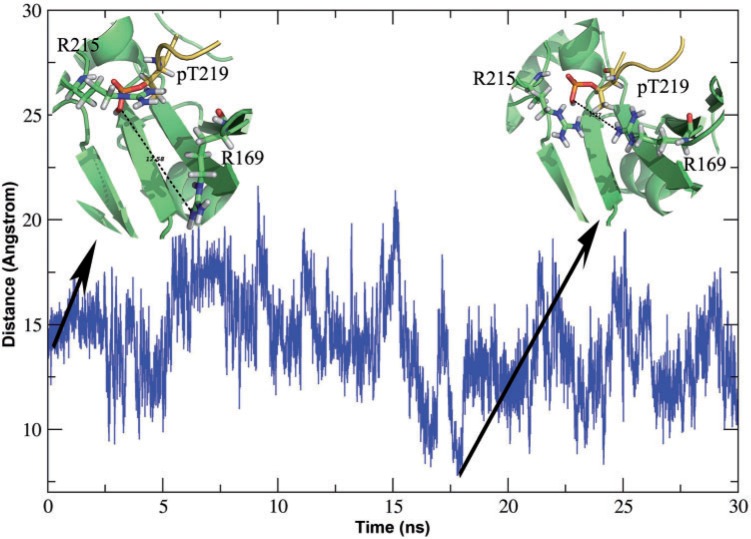
Variation of the distance between R169 and pT219 during MD simulation. The distances have been computed between side chain nitrogen of R169 and oxygen of pT219. The inset shows snapshot taken at the starting of simulation, when distance R169 and pT219 is quite high, that is, 12.58 Å and at point when distance between R169 and pT219 was closest, that is, 7.76 Å. The time point along the trajectory when the snapshots were taken is marked with arrow symbol.

Multiple explicit solvent MD simulations of 30 ns duration were performed on BLES03-peptide complexes to investigate if incorporation of complete flexibilities can bring R169 closer to the pT. MD simulations revealed that ERK peptide was stable in the putative catalytic pocket of BLES03 and R215 that had flipped into the pocket during docking, remained in the catalytic pocket for the entire 30 ns of the MD trajectory. The variation of the shortest distance between the side chain N atoms of R160 and oxygen atoms of the phosphate group of pT over the 30 ns trajectory (fig. 7) revealed that at several time points the distance reduced to 7.8 Å from an initial distance of 13.1 Å. It is possible that R169 moves away from pT because of the electrostatic repulsion between R169 and R215. Thus, MD simulations reveal that upon substrate binding the loop region of BLES03 can adopt a conformation where R169 is in vicinity of catalytic pocket (fig. 7), but due to its higher energy the conformation is not stable. Also, its movement toward catalytic pocket involves rearrangement of secondary structure, which is usually difficult to see during simulations spanning over nanoseconds only. The strong interactions made by positively charged pocket in SpvC locks the ERK peptide in its position and also shield it from solvent for efficient catalysis (Zhu et al. 2007). The average exposed polar surface for pT was 4.43 Å^2^, thus indicating that it is shielded from solvent, even when interaction from R169 is missing. Thus, docking and MD revealed substrate induced conformational changes in BLES03 similar to that of SpvC-like PTL. Molecular dynamic simulations also showed that the pT-containing peptide is stable inside BLES03 pocket, which supports our hypothesis of BLES03 having PTL activity.

### Genome Mining for BLES03 Homolog Reveals Conservation of Putative PTL Catalyzing Residues

**Fig. 8.—:**
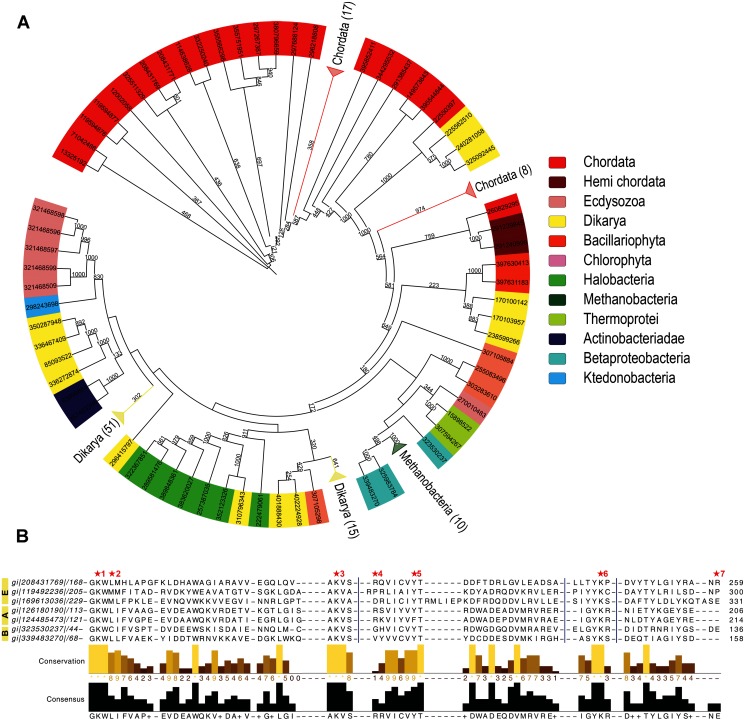
Phylogenetic tree and conservation of putative active site residues of BLES03 homologs. (*A*) Phylogenetic tree of BLES03 homolog. Color on outer ring indicates taxonomic classes of the organism to which the respective protein belongs. To improve clarity, clades containing proteins from same taxonomical classes were collapsed and labeled. The numbers in bracket indicate number of sequences contained in that clade. The numbers next to branches indicate number of time-associated branches clustered together in a bootstrap test with 1,000 replicates. (*B*) Conservation of probable active site residues of BLES03-like proteins in representative sequences obtained from a sequence similarity search using BLAST. Star numbers are same as figures 4 and 5*A*. First three sequences are from eukaryotic organism, next two from archaeabacteria, and the last two from bacteria. For clarity few columns of the MSA were hidden, which is depicted by blue vertical lines.

**Fig. 9.—:**
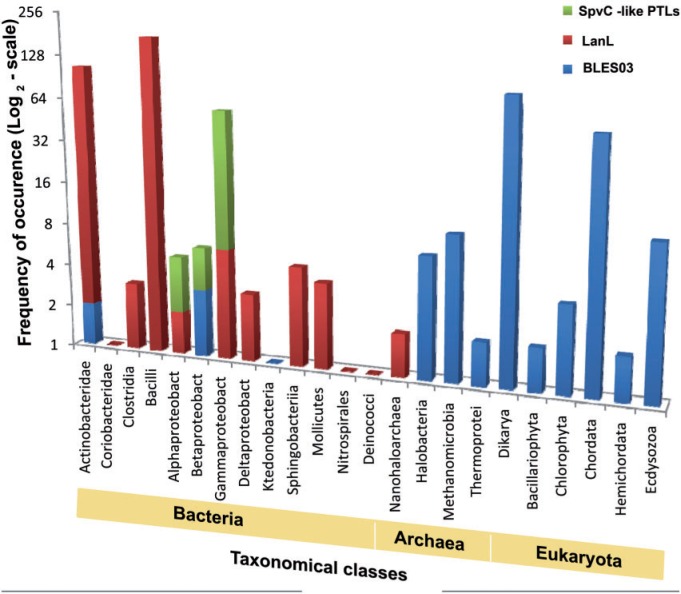
Phylogenetic distribution of eliminylating domains. Spv-C-like PTL (green), VenL-like proteins (red), and BLES03-like proteins (blue). BLES03 proteins are present in bacterial classes which also contain Spv-C-like PTL and VenL-like proteins.

In order to search for BLES03 homologs from all the kingdoms of life, PSI-BLAST search in nr database was carried out using BLES03 as query. Figure 8 shows phylogenetic tree built using hits obtained from PSI-BLAST search. Interestingly, BLES03-like proteins were present in all the three kingdoms of life, that is, bacteria, archaea and eukaryotes (fig. 8*A*; supplementary file S1, Supplementary Material online). Out of the 167 statistically significant hits obtained from the PSI-BLAST search, a majority of BLES03 homologs (142) were from eukaryota, 19 homologs were from archaea, and 6 hits corresponded to bacteria. As can be seen from table 2, all the active site residues of PTL, except R215, are indeed conserved in BLES03 homologs from all three kingdoms of life (fig. 8*B*). Such high degree of conservation among distant homolog provides additional support for our prediction of PTL catalytic function for BLES03. BLES03-like proteins are present in eukaryotes such as chordata as well as dikarya. In addition to eukaryotes, these proteins are also present in archaea such as thermoprotei, halobacteria and methanomicrobia and bacteria classes such as betaproteobacteria and actinobacteridae. Thus, our genome mining analysis reveals that BLES03-like proteins could be phylogenetically distinct class of PTLs which are present in all three kingdoms of life, unlike SpvC- and LanL-like PTLs which are present exclusively in bacteria (fig. 9).

**Table 2:** Percentage Conservation of Putative Active Site Residues of BLES03-Like Proteins across All Genomes and from Archaea

Residue No.	% Conservation (BLAST Result)	% Conservation (in Archaea)
K128	98	100
K159	92	81
R169	51	45
Y175	96	81
K204	99	90
R215	50	—

### Genomic Neighborhood Search Also Suggests PTL Function for Archaeal BLES03 Homolog

**Fig. 10.—:**
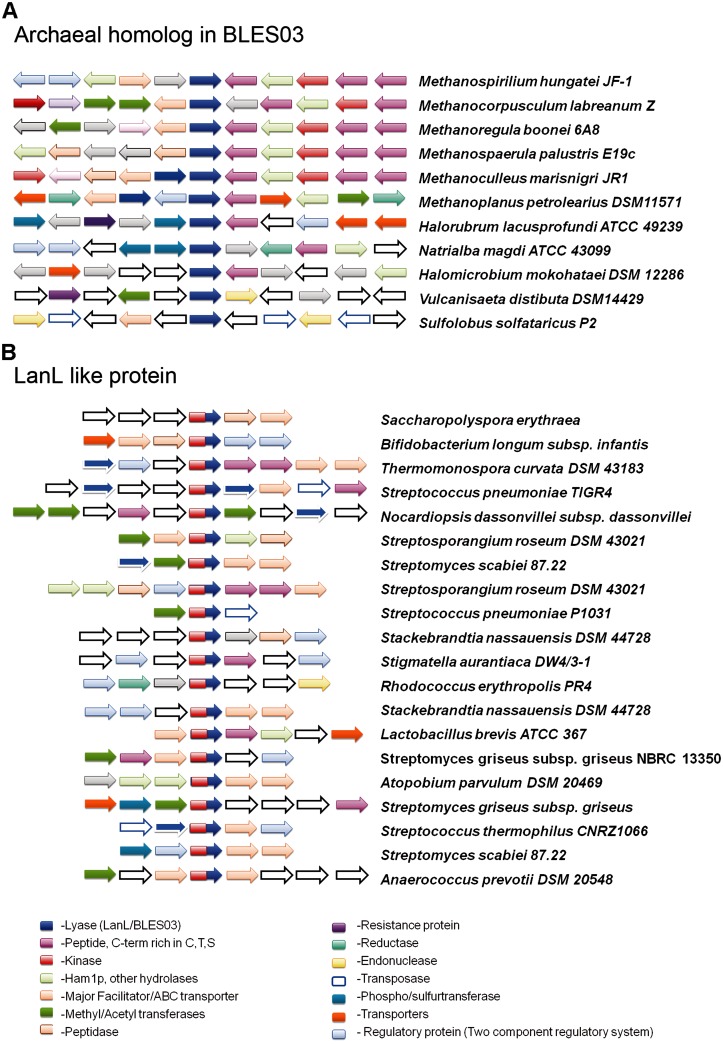
Similar genomic neighborhood of LanL-like proteins and BLES03-like protein in archaea. Synteny of LanL-like proteins (*A*) and BLES03-like protein in archaea (*B*). Each protein is shown as arrows and the source organism is listed on right side. Colors on the arrows have been decoded at the bottom. These annotations have been made only for proteins important in lantibiotic synthesis. BLES03 protein is represented by blue arrows, whereas LanL-like proteins are represented by a combination of red and blue arrow. Cyclization domains from LanL-like protein have not been represented for sake of clarity.

In prokaryotic genomes, it is often found that functionally related genes occur in close proximity to each other on the genome or are fused together (Overbeek et al. 1999). Genomic context analysis has also helped in functional annotation of proteins of unknown function and detection of novel pathways (de Souza and Aravind 2012). Therefore, genome neighborhood of BLES03-like proteins was analyzed and compared with function inferred from our sequence and structure-based analysis. Functional annotation of five upstream and five downstream genomic neighbors of prokaryotic BLES03-like proteins was analyzed using Pfam database (Finn et al. 2014). Genomic neighbors of BLES03-like proteins could be obtained only for 16 cases where complete or partial genome sequences were available (supplementary table S4 in supplementary file S2, Supplementary Material online). It is interesting to note that synteny of 13 archaeal BLES03-like proteins was conserved in terms of presence of other functional domains (fig. 10*A*) and was very similar to lantibiotic gene clusters especially, class IV lantibiotic synthetase (fig. 10*B*). The neighborhood of archaeal BLES03 is usually rich in kinases, hydrolases, peptidase, transport proteins, and at least one small protein whose C-terminal was rich in C, T or S. Similar small proteins rich in C-terminal C, T or S residues found in lantibiotic synthesis pathways are precursors for lanthipeptides. They undergo various PTMs including eliminylation to transform into an active lanthipeptides (fig. 10*B*). Peptidases, also present in the genomic neighborhood of LanL, cleave the N-terminal signal sequence generating a mature lanthipeptide which is then transported out of the cell by transport proteins.

Genes encoding two-component regulatory proteins, atypical kinase Rio1 fused with peptidase, methyltransferase, and acetyltransferase were also found in the genomic neighborhood of archaeal BLES03-like proteins. Analogous proteins are present in the genomic neighborhood of lantibiotic synthetases (Altena et al. 2000). To coordinate lantibiotic production with other cellular events, lantibiotic gene clusters are under regulation of two-component systems. A number of modifications other than dehydration and cyclization are also performed by different tailoring enzymes present in these gene clusters. Examples of these tailoring enzymes are oxidative decarboxylase, dehydrogenase, and hydroxylase (Willey and van der Donk 2007). Also several methyltransferases and acetyltransferases are found in class I lantibiotic gene clusters (Marsh et al. 2010). Thus, our genomic neighborhood analysis revealed that archaeal BLES03 and kinases in its synteny are functional counterparts of lyase domain containing LanL-like proteins in bacteria. These archaeal BLES03-like proteins might be catalyzing PTL activity and could be involved in synthesis of lanthipeptide-like natural products. These gene clusters could be a new class of lantibiotic synthetase clusters, which are yet to be explored.

Analysis of context dependent features provides additional and independent evidence in support of our prediction of PTL activity for BLES03. These evidences suggest that these three classes of proteins have a common ancestor but have evolved through sequence divergence (supplementary fig. S4 in supplementary file S2, Supplementary Material online) to occupy all three kingdoms of life.

### Independent Literature Evidences in Support of the Role of BLES03 as PTL

Even though BLES03 is an acronym for basophilic leukemia expressed protein 03, as of today there is no information available for the precise molecular function of this protein. In view of our bioinformatics prediction of PTL function for BLES03, we wanted to search for literature-based evidence in support of putative PTL activity of BLES03. Pathogenic SpvC-like lyases are known to affect Thr phosphorylation. Therefore, evidences supporting association of BLES03 with protein phosphorylation pathways were searched. Though we could not find any published data which show overexpression of BLES03 in basophilic leukemia, it was interesting to note that in RBL-2H3.2 cell line (low releasing variant of Rat Basophilic Leukemia) Thr phosphorylation by Protein Kinase C δ (PKCδ) on γ chain of FcεR1 is delayed and the same variant has defect in PKC activity (Bingham et al. 1994). Hence, it is tempting to speculate that Thr eliminylation activity of BLES03 could be the reason for the defect in PKC activity. Also, analysis of expression data on 60 different cancer cell lines (Ross et al. 2000) showed that BLES03 is expressed in many cancer cell lines and in seven such cases it is among top 5% of all genes ranked by expression levels. These evidences suggest that BLES03 has a cancer-associated expression. Our prediction of BLES03 as eukaryotic PTL could provide clues to design experiments for better functional characterization of BLES03-like proteins in multicellular eukaryotes.

Apart from the involvement in regulation of signaling pathways, in multicellular eukaryotes BLES03 could also function in metabolic pathways involving dehydro amino acids similar to LanL-like PTLs. Dhas can be produced from cysteine, selenocysteine, and phosphoserine (pS) residues in a protein and their presence in proteins from different human tissues has been hypothesized (Cooper et al. 2011). Dha and Dhb produced from pS and pT, respectively, have been identified in aged and cataractogenous human lenses (Linetsky et al. 2004), though mechanistic details of their biosynthesis are unknown. Also, it is known that dehydro residues undergo irreversible glutathionylation by addition of GSH to form lanthionines (Skolnick et al. 2000; Bhattacharyya et al. 2002; Punta et al. 2012). LanCL1, human homolog of prokaryotic lanthionine synthase-C (LanC), was demonstrated to be a novel glutathione-binding protein (Chung et al. 2007). Also, active site residues of LanC are conserved in LanCL1. Armed with these findings Chung et al. have hypothesized that LanCL1 might have an activity analogous to bacterial LanC, wherein LanCL1 is involved in formation thioether linkages using GSH as one substrate. The second substrate of LanCL1 for formation of thioether linkages using GSH might be Dha/Dhb. In view of these literature evidence, it is possible that BLES03 might act upstream of LanCL1 converting phophothreonine/serine to Dha/Dhb, respectively (supplementary fig. S5*A* in supplementary file S2, Supplementary Material online). These dehydro residues might then be acted upon by LanCL1 to convert them to a glutathione adduct. A recent study has demonstrated that LanCL1 binds and inhibits cystathione β-synthase (CBS) (Krishnadev and Srinivasan 2011). It was also found that GSH/GSSG ratio in cells controlled LanCL1-CBS binding and hence its activity, though the mechanism could not be established. Based on our bioinformatics analysis, one can hypothesize that BLES03 might act on a Thr/Ser residue of CBS converting it to Dhb/Dha which can then undergo LanCL1 catalyzed Michael addition with GSH to form glutathione–CBS adduct (supplementary fig. S5*B* in supplementary file S2, Supplementary Material online). This unusual PTM might inhibit CBS. Thus, literature evidences suggest presence of Dha/Dhb, lanthionines, and LanC-like enzymes, which are key components of lanthipeptide biosynthetic pathways in multicellular eukaryotes. Our structure- and genomic context-based bioinformatics analyses suggest that the missing link of eukaryotic PTL could be played by BLES03 and biosynthetic pathways similar to lantipeptide biosynthetic pathways could indeed be present in multicellular eukaryotes including humans.

## Discussion

Eliminylation is a novel PTM catalyzed by newly discovered enzyme family called PTLs. PTLs were first discovered in pathogenic bacteria such as *Sh. flexneri*, *Pseudomonas syringae* and *Sa. enterica* and subsequently, in lantibiotic producing bacteria such as *Streptomyces venezuelae* and *Lactococcus lactis* subsp. *lactis*. Even though there is no obvious sequence similarity between SpvC-like PTLs and LanL-like PTLs, their catalytic mechanisms are strikingly similar. They work in close coordination with Ser/Thr kinases to posttranslationally modify Ser or Thr residues to Dha or Dhb, respectively. As several experimental studies have identified dehydro amino acids such as Dha/Dhb in eukaryotes including humans, it has been contemplated that PTLs might also be present in eukaryotes. However, even sensitive profile-based sequence comparisons have failed to detect any functional analogs of PTLs in eukaryotes. As protein structure is evolutionarily more conserved than sequence, in cases where conventional sequence-based homology searches fail to identify putative homologs, comparison of fold level structural similarity in combination with chemical similarity in the active site has helped in identification of proteins with similar catalytic functions (Skolnick et al. 2000; Bhattacharyya et al. 2002). Inspired by these reports, we carried out structure-based genome-wide search for eukaryotic PTL domains.

Our fold level structural similarity search and comparison of similarities in putative active site pockets helped in detection of eukaryotic protein BLES03, a human protein with unknown function, as functional counterpart of PTLs. Structural similarity between BLES03 and eIF4-E has been reported earlier (Bitto et al. 2005; Andreeva et al. 2008) but essential catalytic residues of eIF4-E were found missing in BLES03. However, based on the presence of a basic surface patch having electrostatic surface potential similar to eIF4-E, Bitto et al. (2005) have hypothesized that BLES03 might be involved in nucleic acid binding. In this work, we show that this basic patch of BLES03 contains key residues required for stable binding of pT-containing peptide. Similar to SpvC-like PTLs, upon substrate binding there is a conformational change in the loop region which facilitates movement of crucial arginines into the catalytic pocket and assembly of complete pocket for catalyzing eliminylation reaction. Though BLES03 shares a higher degree of overall structural similarity with eIF4E, a more detailed active site comparison involving docking and MD simulations suggests BLES03 to be functionally analogous to PTLs (supplementary fig. S2 in supplementary file S2, Supplementary Material online). Interestingly, the crystal structure of BLES03 shows the putative pT-binding pocket to be interacting with N-terminal helix of symmetry-related monomer (supplementary fig. S3 in supplementary file S2, Supplementary Material online). It is possible that this N-terminal helix plays a regulatory role by occluding the active site of BLES03 under normal conditions and making it catalytically inactive. In disease conditions, the movement of this N-terminal helix makes the catalytic site accessible and BLES03 functions as a PTL. Similar regulatory mechanism has been demonstrated in case of the human protein HYPE which shares structural and functional similarity with AMPylating enzymes found in pathogenic bacteria (Engel et al. 2012).

In addition to overall fold and catalytic site similarity, we also provide several other lines of evidence to conclude an evolutionary link between SpvC- and LanL-like PTL and BLES03. A search for homologs of BLES03 across all kingdoms of life confirmed its presence in eukaryotes, bacteria, and archaebacteria. The catalytic and binding pocket residues identified by our structure-based search were also found to be evolutionarily conserved. The analysis of genomic neighborhood of BLES03-like protein in archaea showed striking similarity with class III and IV lantibiotic synthetase gene clusters. The similarity in synteny of archaeal homolog of BLES03 and LanL-like PTL is independent evidence which suggests PTL function for BLES03. Our genome-wide search has also identified lantipeptide clusters where kinase and PTL are present as standalone proteins unlike class III and IV lantibiotic synthetases, where they occur as fused domains on a single polypeptide. Therefore, combining sequence analysis and structural comparison with classical phylogenetic tree analysis we have shed light on the evolutionary history of eliminylating domains.

In summary, the structure-guided in silico genome mining study has revealed that BLES03-like proteins are putative PTLs and are involved in biosynthesis of dehydro amino acids in eukaryotes and lanthipeptide secondary metabolites in archaebacteria. The computational predictions presented in this work would provide valuable inputs to design experiments for identification of eukaryotic PTLs and discovery of new lanthipeptide gene clusters.

## Supplementary Material

Supplementary files S1–S5 are available at *Genome Biology and Evolution* online (http://www.gbe. oxfordjournals.org/).

Supplementary Data
