# Supplementary material for: Genome-Wide Search for Eliminylating Domains Reveals Novel Function for BLES03-Like Proteins
Source: Genome Biol Evol. 2014 Jul 24;6(8):2017–33. doi: 10.1093/gbe/evu161 (PMC4159009; doi:10.1093/gbe/evu161)
Supplement: Supplementary Data [file supp_6_8_2017__index.html]

Genome wide search for eliminylating domains reveals novel function for BLES03 like proteins — Genome-Wide Search for Eliminylating Domains Reveals Novel Function for BLES03-Like Proteins — Supplementary Data 

# Genome-Wide Search for Eliminylating Domains Reveals Novel Function for BLES03-Like Proteins

## Supplementary Data

files

**Files in this Data Supplement:**

- Supplementary Data - zip file
