# Supplementary material for: Genome-Wide Search for Eliminylating Domains Reveals Novel Function for BLES03-Like Proteins
Source: Genome Biol Evol. 2014 Jul 24;6(8):2017–33. doi: 10.1093/gbe/evu161 (PMC4159009; doi:10.1093/gbe/evu161)
Supplement: Supplementary Data [file supp_evu161_suppl_data.zip › Additional File 2.doc]

**Supplementary Table S1**:

| **Query** | **BLAST** | **PSI-BLAST** |
| --- | --- | --- |
| SpvC (Phosphothreonien lyase) | 53 | 55 |
| VenL (LanL lyase) | 250 | 301 |

S**upplmentary Table S2**: HHPred results using target database as PfamA and PDB70

A) PfamA (Hits with e-value less than 1 reported)

| **Query** | **Rank** | **Pfam Family ID** | **Probability** | **E-value** | **Query HMM** | **Template HMM** |
| --- | --- | --- | --- | --- | --- | --- |
| SpvC | 1 | PF03536 | 100 | 3.00E^-^136 | 2 to 241 | 1 to 240 |
|  |  |  |  |  |  |  |
| VenL | 1 | PF03536 | 93.1 | 0.2 | 25 to 149 | 73-212 |

B) PDB70 (Only Top Hits reported)

| **Query** | **Rank** | **PDB ID** | **Probability** | **E-value** | **Query HMM** | **Template HMM** |
| --- | --- | --- | --- | --- | --- | --- |
| SpvC | 1 | 2P1W (Phosphothreonine lyase) | 100 | 1E-132 | 1 to 240 | 11 to 250 |
|  |  |  |  |  |  |  |
| VenL | 1 | 3IOU (Phosphothreonine lyase) | 94 | 0.34 | 25-149 | 51 to 190 |

**Supplementary Table S3:**

A) Pfam (Hits with e-value less than 1 reported)

| **Query** | **Rank** | **PDB ID** | **Probability** | **E-value** | **Query HMM** | **Template HMM** |
| --- | --- | --- | --- | --- | --- | --- |
| BLES03 | 1 | PF08939  (BLES03) | 100 | 9E-57 | 20-212 | 26-243 |
|  | 2 | PF01652  (eIF4E) | 95.9 | 0.013 | 122-205 | 77-164 |

B) PDB (Hits with e-value less than 1 reported)

| **Query** | **Rank** | **PDB ID** | **Probability** | **E-value** | **Query HMM** | **Template HMM** |
| --- | --- | --- | --- | --- | --- | --- |
| BLES03 | 1 | 1ZTP (BLES03) | 100 | 1E-70 | 1-193 | 20-212 |
|  | 2 | 2IDR  (eIF4E) | 97.2 | 0.00099 | 108-186 | 79-160 |

**Supplementary Table S4: List of BLES03 containing archaeabacterial genomes**

List completely or partially sequenced archaeabacterial genomes where BLES03 was found using PSI-BLAST. Those genomes are marked as bold where synteny of BLES03 was conserved

| **Gene Identifier (GI) from NCBI Protein** | **Source organism** |
| --- | --- |
| **355571464** | ***Methanolinea tarda NOBI-1*** |
| **289581476** | ***Natrialba magadii ATCC 43099*** |
| **124485473** | ***Methanocorpusculum labreanum Z*** |
| **219851257** | ***Methanosphaerula palustris E1-9c*** |
| **257387035** | ***Halomicrobium mukohataei DSM 12286*** |
| **126180190** | ***Methanoculleus marisnigri JR1*** |
| **88604104** | ***Methanospirillum hungatei JF-1*** |
| **222479061** | ***Halorubrum lacusprofundi ATCC 49239*** |
| **154149784** | ***Methanoregula boonei 6A8*** |
| **307352281** | ***Methanoplanus petrolearius DSM 11571*** |
| **374629092** | ***Methanoplanus limicola DSM 2279*** |
| **307594267** | ***Vulcanisaeta distributa DSM 14429*** |
| **15898522** | ***Sulfolobus solfataricus P2*** |
| 352123326 | *Natronobacterium gregoryi SP2* |
| 322367851 | *Haladaptatus paucihalophilus DX253* |
| 383620027 | *Halobiforma lacisalsi AJ5* |

**Supplementary Figure S1: Docking of ERK peptide on BLES03 structure**

(A) Conformational changes similar to SpvC is seen in Bles03 on docking. The figure shows R215 moving in close proximity to pT of ERK peptide.

(B) Docked peptides from top ten clusters of HADDOCK result. All peptides occupy the proposed catalytic binding pocket.

**Supplementary Figure S2**: Based on PSI-BLAST score (cut off e-03) eIF4-E (pink), BLES03 (blue), phosphothreonine lyase (yellow) and LanL-like (green) clusters were visualized using cytoscape and it was seen that they do not share any sequence similarity (blue lines). But structural similarity (black arrows, numbers are DALILITE Z- score) suggest eIF4-E, BLES03, phosphothreonine lyase to be homologous. Red arrows indicate a connecting link when both active site residues and secondary structures were conserved. Hence though eIF4-E, BLES03, phosphothreonine lyase LanL-like proteins forms 4 different closely knit clusters based on sequence information, structural information provides link between all four.

**Supplementary Figure S3:** Cartoon representation depicting T21 from N-terminal helix of symmetry related BLES03 monomer (chain B) occupies the same position as pT from docked ERK peptide.

**Supplementary Figure 4: Evolutionary relationship between the four families.** Phylogenetic tree built using representative sequences of LanL, phosphothreonine lyase (PTLs), BLES03 and eIF4E. The leaf labels of the phylogenetic trees are colored based on their family and the branches are colored green (high) to red (low) based on their bootstrap values.

**Supplementary Figure 5:** Probable pathways where BLES03 and LanCL1 might be involved in humans based on literature evidence
